# Racial/ethnic differences in mental health treatment received among people with comorbid cardiometabolic and depressive symptomology: National Health and Nutrition Examination Survey, 2017 to March 2020 Pre-Pandemic

**DOI:** 10.1371/journal.pone.0316430

**Published:** 2025-01-02

**Authors:** Tyra Dark, Rachel Harris, Desiree Burns, Jacob Chernicky, Laura Reid-Marks, George Rust

**Affiliations:** 1 Department of Behavioral Sciences and Social Medicine, College of Medicine, Florida State University, Tallahassee, Florida, United States of America; 2 College of Social Work, Florida State University, Tallahassee, Florida, United States of America; 3 Department of Psychology, Florida State University, Tallahassee, Florida, United States of America; Complete HEOR Solutions, UNITED STATES OF AMERICA

## Abstract

**Background:**

Individuals with chronic physical conditions and comorbid mental illness have increased probability of adverse health outcomes. As minority populations have limited access to both medical care and culturally appropriate mental health services, having a comorbid mental health condition can further impede their ability to manage chronic conditions and widen racial disparities in health outcomes. Further, racial/ethnic disparities in treatment patterns are likely to exacerbate disparities in adverse health outcomes.

**Objective:**

To identify the racial/ethnic mental health treatment patterns among individuals with cardiometabolic and depressive symptomology co-occurrence.

**Methods:**

This study utilized National Health and Nutrition Examination Survey data, 2017 to March 2020 Pre-Pandemic. The primary analysis was an adjusted linear logistic regression analysis of race/ethnicity, comorbidity status and mental health treatment type. Regression models were estimated to determine the likelihood of receiving counseling and medication therapy, and to determine if the likelihood is associated with race/ethnicity.

**Results:**

Primary findings indicate that depressive symptomology only was the most common designation and fewer than half of persons received any mental health treatment. Across all racial/ethnic groups, receiving no mental health treatment was the most common designation. Sixty-one percent of Non-Hispanic White persons and more than three out of four Hispanic and Non-Hispanic Black persons with only depressive symptoms received no mental health treatment. Adjusted regression analyses revealed that participants with comorbid cardiometabolic and depressive symptomology have 28% lower odds of receiving combined mental health professional and medication therapy than participants with depressive symptomology only.

**Conclusions:**

Simultaneously treating both mental illness and cardiometabolic symptoms properly is complicated, but there may be untapped synergies in treating both concurrently. Therefore, to achieve favorable health outcomes, policy should be implemented to optimize clinical treatment by addressing aspects of both conditions in an integrated approach and may need to be culturally tailored to be effective.

## Introduction

Cardiometabolic syndrome (CMetS) and depressive disorders (DD) are prevalent, co-occur, and are unequally distributed across racial/ethnic and economic population groups [1]. As co-occurring chronic conditions, they raise significant barriers to effective comprehensive disease management and result in more adverse outcomes [2, 3]. The prevalence of CMetS varies substantially across different racial/ethnic, gender, and age groups in the U.S. population [4]. The overall prevalence of CMetS rose by more than a third from 1988–1994 to 2007–2012, increasing from 25.3% to 34.2% [5]. The largest increase in the prevalence of CMetS was observed among non-Hispanic black men (55%), then non-Hispanic white women (44%), and non-Hispanic black women (41%). Similar increasing trends in the prevalence of depressive disorders have been observed in the US. There are documented increases in depression prevalence among youth, adolescents, and adults 60 and over [6, 7]. Although twelve-month prevalence of major depressive episodes has increased among adolescents and young adults from 2005 to 2014 [7], the magnitude of the increasing trend was even greater in populations aged ≥60 years [6].

Depression itself has been identified as a cardiometabolic risk factor, independent of other known risk factors. In a 2013 study, Medicare beneficiaries with depression were twice as likely as those without depression to develop diabetes [8]. Depression is associated with a 15% increased risk of cardiovascular disease [9]. While a clear link between treatment of depression and reduction of adverse cardiovascular events has not been established, the under-diagnosis and under-treatment of depression could lead not only to worsening of other known cardiac risk factors in the constellation of CMetS, but could also impede the effective medical and behavioral management of these risk factors [10]. Depression has previously been documented to be both under-diagnosed and under-treated, [7, 11]—largely attributed to a shortage of outpatient mental health services, health plan barriers, clinical inertia, social stigma, cultural differences, and other personal inhibitions [12, 13].

Individuals with chronic physical conditions and comorbid mental illness have increased probability of adverse health outcomes [2, 3] and poorer quality of life [14]. Gaulin et al. found that in 2014–15, “the synergy between mental and physical disorders accounted for … 5.8% of all emergency department visits across the province” [of Quebec]. A recent review found that cardiovascular disease and depression are *“mutually causative and exert reciprocal effects on one another”* [15].

Multimorbidity is increasing in prevalence nationally, with racial-ethnic disparities persisting for at least the last two decades [16]. Using national Health and Retirement Survey data, Quinones et al. found that African Americans not only have higher rates of multimorbidity, but also develop multiple chronic conditions an average of four years earlier than do white non-Hispanic persons [17]. They also found that Hispanic persons accumulated chronic conditions at a rate 60% faster than did non-Hispanic white subjects. Higher education levels were somewhat protective.

For African American (AA) and Hispanic / LatinX populations, who more often have limited access to both medical care and culturally appropriate mental health services compared to White non-Hispanic patients, having a comorbid mental health condition can further impede their ability to manage chronic medical conditions and widen racial disparities in health outcomes. To effectively address comorbid CMetS and depression, it is vital to identify culturally specific points of intervention on the care continuum to lessen disproportionate burden of disease among vulnerable populations. Therefore, we undertook this research in order to identify the racial/ethnic mental health treatment patterns among individuals with CMetS and Depression co-occurrence in a nationally representative sample. Specifically, the objective of this study was to document utilization of two forms of treatment (mental health counseling and/or medication therapy) of depression among racial/ethnic groups with co-occurring depression and CMetS.

## Methods

### Data

Ethics Statement. This study utilized National Health and Nutrition Examination Survey (NHANES) data. The NHANES study was approved by the National Center for Health Statics Ethics Review Board, and no external ethic approval was required for this current study. This study was exempt from review by the Institutional Review Board of Florida State University.

The NHANES surveys have been conducted annually by the National Center for Health Statistics since 1999, using a complex multistage sampling design to obtain a representative sample of the civilian, noninstitutionalized US population [18]. The NHANES oversamples minorities and allows for population estimates using population totals from the Current Population Surveys. To obtain an adequate sample size for the analyses we utilized data from the 2017 to March 2020 Pre-Pandemic NHANES, for a potential total sample size of 18,248 adults aged 18 and older.

#### Study design and study population

A cross-sectional analysis of 2017 to March 2020 Pre-Pandemic NHANES data was performed. Survey respondents for which there is a reporting and determination of (1) depressive disorder (DD) symptoms and (2) cardiometabolic syndrome (CMetS) risk factors were included in the study. CMetS was assessed using the following criteria: (1) Reporting a type-2 diabetes diagnosis AND (2) reporting at least two of three conditions—hypertension, obesity, or hyperlipidemia. Depressive symptoms were assessed using the PHQ-9, a 9-item screening tool that asks participants to choose 1 of 4 responses about the frequency of depressive symptoms during the previous 2 weeks [19]. A score of 10–14 was categorized as moderate, a score of 15–19 was considered moderately severe, and a score of 20 or greater was considered severe depressive symptoms.

### Study variables

#### Outcome variable

The outcome for this study is treatment type. Treatment type was categorized into the following: (1) Medication use, (2) psychological counseling and therapy, (3) combined medication and psychological counseling and therapy, and (4) no treatment. Medication use was defined as taking at least one prescribed medication in the past 30 days for depressive symptomology. During the household interview, survey participants were asked if they had taken a medication in the past month for which they needed a prescription. Those who answered “yes” were asked to show the interviewer the medication containers of all the medications used. Counseling and therapy. Although the NHANES does not provide details on psychological counseling, we defined counseling and various types of therapy as treatment with a mental health professional, which was measured by the survey question, “During the past 12 months, have you seen or talked to a mental health professional such as a psychologist, psychiatrist, psychiatric nurse, or clinical social worker about your health?” Because evidenced-based treatment recommendations for prescribing antidepressant medication and/or administering psychotherapy exist for individuals with PHQ-9 scores >5, we specifically examined all forms of treatment among respondents that scored >5 on the PHQ-9.

#### Independent variable

The primary independent variable was race/ethnicity. The four race-ethnicity groups included: (1) non-Hispanic white, (2) non-Hispanic black, (3) Mexican American, and other Hispanic, and (4) other races.

#### Control variables

The analyses included independent variables potentially associated with mental health treatment type. We adjusted the logistic regression model to control for the following variables: age, sex, educational attainment, and insurance type. Respondents used in this analysis were limited to those aged 18 and over. Age was included in the analysis as a categorical variable with the following 3 groups: 18–39, 40–59 and 60 and over. Educational attainment was categorized as (1) less than high school degree, (2) high school graduate or GED, (3) some college or associate degree, and (4) college graduate or above. Insurance status was categorized as (1) not insured, (2) private, (3) Medicaid, (4) Medicare, and (5) other State-sponsored plans (not including CHIP, Medicare, Medicaid).

### Statistical analysis

All data analyses were performed by using the survey procedures of SAS software, version 9.4. Frequencies, weighted population estimates, standard errors, and 95% CIs taking into account the complex sampling design and population weights were generated by Proc Crosstabs in SAS version 9.4 (Research Triangle Institute, Research Triangle Park, NC). First, overall prevalence of depressive symptoms and prevalence of the different depressive symptom severity categories were assessed for the entire adult population. Treatment (mental health professional and/or antidepressant) use by depressive symptom severity was then assessed. Prevalence of depressive symptoms, depressive symptom severity, and treatment among different racial/ethnic groups was also examined. Descriptive statistics were also calculated for participants reporting CMetS risk factors—with no depressive symptomology—and participants reported depressive symptoms—with no CMetS risk factors.

The primary analysis was an ordinal logistic regression analysis of race/ethnicity, comorbidity status (CMetS and/or depressive symptomology) and mental health treatment type. Logistic regression models were estimated to determine the likelihood of receiving mental health related treatment (talk and medication therapy) and to determine if the likelihood is associated with race/ethnicity. Adjusted models controlled for comorbid conditions, age, sex, educational attainment, and insurance type. The SURVEYLOGISTIC procedure in SAS fits logistic regression models for discrete response survey data by the method of maximum likelihood and incorporates the sample design into the analysis to enable results to be nationally representative and for standard errors to correctly account for the stratified random sample. In each regression analysis, we present odds ratios with p-values.

## Results

Descriptive statistics for the study population (n = 9693) are included in Table 1. Among the total sample, 695 (5.53%) participants reported CMet risk factors—with no depressive symptomology—and 1949 (20.63%) participants reported depressive symptoms—with no CMet risk factors. Comorbid CMet risk and depressive symptoms was present for 337 (2.52%) participants. Although, most study participants reported no mental health treatment (n = 8290, 83.69%), receiving treatment from a mental health professional only was the most commonly reported type of treatment (n = 685, 7.84%).

**Table 1:** Frequency of sample demographics and study variables.

	Frequency	Weighted Frequency	Std Err of Wgt Freq	Percent	Std Err of Percent
**Race**					
**Non-Hispanic White**	3370	154162965	3192023	62.1758	0.6599
**Non-Hispanic Black**	2555	28420787	600078	11.4624	0.2957
**Hispanic**	2122	40485465	965050	16.3283	0.4302
**Other race**	1646	24877844	799394	10.0335	0.3402
**Age**					
**18–39**	3260	94125616	2169496	37.9620	0.7544
**40–59**	3011	82164387	2203824	33.1379	0.7554
**60+**	3422	71657057	1784350	28.9001	0.6762
**Gender**					
**Male**	4718	119592918	2466203	48.2332	0.7790
**Female**	4975	128354142	2372811	51.7668	0.7790
**Insurance**					
**No insurance**	1554	32892008	1128585	13.9484	0.4798
**Private**	3560	116534231	2719523	49.4183	0.8021
**Medicare**	2065	46375198	1396581	19.6662	0.5808
**Medicaid**	1555	28079999	1004911	11.9078	0.4317
**Other state sponsored**	447	11930698	814219	5.0594	0.3409
**Educational Attainment**					
**Less than High School**	2221	34035737	928214	13.7409	0.4022
**High School Grad/GED**	2225	64776211	1944653	26.1515	0.7026
**Some College**	2975	73170401	1797785	29.5404	0.6813
**College graduate+**	2257	75713868	2303652	30.5672	0.7748
**Conditions**					
**Cardiometabolic Only**	695	13712935	819928	5.5306	0.3278
**Cardiometabolic and Depressive**	337	6244097	572677	2.5183	0.2298
**Depressive Only**	1949	51145551	1660313	20.6276	0.6263
**Neither**	6712	176844477	2758792	71.3235	0.6929
**Treatment Type**					
**No Treatment**	8290	207499659	2795307	83.6871	0.5940
**Mental Health Counseling**	685	19445045	1148853	7.8424	0.4486
**Medication only**	397	11618816	798972	4.6860	0.3186
**Counseling and Medication**	321	9383540	781983	3.7845	0.3111

Table 2 demonstrates that health conditions were not equally distributed across racial/ethnic groups (*X*^2^ (9) = 28.16; p < .0001). The highest percentage of each racial/ethnic group reported having neither health condition. Among all racial/ethnic groups, demonstrating depressive symptomology only (no cardiometabolic risk factors) was the most common designation. Having comorbid CMetS and depressive symptomology together was the least common designation. Having cardiometabolic risk factors only occurred most often for non-Hispanic Black (6.86%; SE = 0.53), followed by non-Hispanic White (5.43%; SE = 0.48), Mexican American and Other Hispanics (5.18%; SE = 0.50), and other Races (5.18%; SE = 0.80). Prevalence of only depressive symptoms was highest among Mexican Americans and Other Hispanics (23.20%; SE = 1.10), followed by non-Hispanic Whites (20.81%; SE = 0.93), non-Hispanic Black (19.63%; SE = 0.94), and Other Races (16.42%; SE = 1.28). The prevalence of comorbid cardiometabolic risk and depressive symptoms was highest among non-Hispanic Black participants (3.05%, SE = 0.33) compared to all other racial/ethnic groups.

**Table 2:** Frequency of cardiometabolic risk factors and depressive symptomology by race/ethnicity in the study population.

	Non-Hispanic White	Non-Hispanic Black	Mexican American and Other Hispanic	Other Race and Multi Race
	(n = 154,162,965)	(n = 28,420,787)	(n = 40,485,465)	(n = 24,877,844)
Cardiometabolic Risk Factors Only				
Frequency	8,377,124	1,950,790	2,096,690	1,288,332
Percent (Std Error)	5.43 (0.48)	6.86 (0.53)	5.18(0.50)	5.18 (0.80)
Depressive Symptomology Only				
Frequency	32,088,241	5,580,314	9,392,341	4,084,655
Percent (Std Error)	20.81 (0.93)	19.63 (0.94)	23.20 (1.10)	16.42 (1.28)
Cardiometabolic & Depressive Symptomology				
Frequency	3,883,338	867,871	826,051	666,837
Percent (Std Error)	2.52 (0.34)	3.05 (0.33)	2.04 (0.30)	2.68 (0.70)
Neither				
Frequency	109,814,262	20,021,812	28,170,383	18,838,020
Percent (Std Error)	71.23 (1.03)	70.45 (1.05)	69.58 (1.17)	75.72 (1.52)

*X*^2^ (9) = 28.16; p < .0001

Table 3 demonstrates that treatment type was not equally distributed across racial/ethnic groups for those individuals with depressive symptomology only (*X*^2^ (9) = 35.82; p < .001). Among those with any level of depression, 7,226,484 (14.13%) received only mental health professional counseling, 4,413,991 (8.63%) received only medication treatment, and 5,804,056 (11.35%) received both mental health professional counseling and medication treatment. Across all racial/ethnic groups, receiving no mental health treatment was the most common designation. Overall, fewer than half of persons with depressive symptoms received any treatment at all. More than one in three were untreated even among those with moderately severe (46.28% untreated) or severe depression (36.51% untreated). Further, Non-Hispanic Black, and Mexican American and other Hispanic participants demonstrated the highest prevalence of no mental health treatment received (75.56% and 75.0%, respectively). A pattern emerged across all racial/ethnic groups where the highest percentage of participants received mental health professional only therapy, followed by the combination of both mental health professional and medication therapy. Rates for treatment in the form of a combination of both mental health professional and medication therapy were highest among non-Hispanic White at 13.45%, and lowest among Non-Hispanic Blacks, and Mexican American and other Hispanics at 7.22% and 7.00%, respectively.

**Table 3:** Weighted frequency (standard errors) of mental health treatment type presented by race/ethnicity and health conditions.

**Participants with Depressive Symptomology Only**					
**Treatment Type**	**Non-Hispanic White**	**Non-Hispanic Black**	**Mexican American and Other Hispanic**	**Other Races**	**Total**
No Mental Health Treatment	19,641,143 (1182039)	4,105,208 (238207)	7,044,615 (404171)	2,910,054 (266775)	**33701020** **(1158427)**
Mental Health Professional Only	4,525,766 (600417)	852,477 (111118)	1,256,873 (199127)	591,368 (144410)	**7226484** **(646856)**
Medication Only	3,605,509 (466192)	219,756 (61890)	433,934 (104947)	154,793 (57268)	**4413991** **(481938)**
Mental Health Professional and Medication	4,315,823 (597408)	402,873 (84206)	656,919 (133034)	428,441 (153766)	**5804056** **(630870)**
**Total**	**32,088,241**	**5,580,314**	**9,392,341**	**4,084,655**	**51145551**
Values provided as n (%). *X*^2^ (9) = 35.82; p < .0001					
**Participants with Cardiometabolic and Depressive Symptomology**					
**Treatment Type**	**Non-Hispanic White**	**Non-Hispanic Black**	**Mexican American and Other Hispanic**	**Other Races**	**Total**
No Mental Health Treatment	2,122,315 (404702)	503,338 (66574)	459,355 (86910)	261,798 (72499)	**3346806** **(403336)**
Mental Health Professional Only	555,879 (201668)	123,699 (34269)	141,369 (47313)	167,793 (134476)	**988740** **(245694)**
Medication Only	876,849 (217551)	140,372 (37888)	149,928 (45386)	155,979 (80146)	**1323129** **(233502)**
Mental Health Professional and Medication	328,295 (95759)	100,461 (34874)	75,399 (43981)	81,267 (34674)	**585422** **(113534)**
**Total**	**3,883,338** (482705)	**867,871** (83095)	**826,051** (111923)	**666,837** (173269)	**6244097** (465723)
Values provided as n (%). *X*^2^ (9) = 3.42; p = 0.948					

Among participants with CMetS and depressive symptomology, treatment type was not unequally distributed across racial/ethnic groups (*X*^2^ (9) = 3.42; p = 0.948) and the majority of the participants did not receive any mental health treatment for their symptoms (see Table 3). For this population with both comorbid CMetS and depressive symptoms, 988,740 (15.83%) received only mental health professional counseling, 1,323,129 (21.19%) received only medication treatment, and 585,422 (9.38%) received both mental health professional counseling and medication drug treatment. The Other race participants were more likely to receive mental health treatment across all three mental health treatment types. Non-Hispanic Black participants reported a lower prevalence of treatment from either a medical professional only or receive medication only compared to other racial ethnic groups. Non-Hispanic participants had a higher prevalence of no mental health treatment compared to other racial ethnic groups.

### Multivariate ordinal logistic regression

The probability of receiving combined mental health professional counseling and medication therapy was modeled in an ordinal logistic regression. Race/ethnicity was the primary predictor variable, and the model was adjusted for comorbidity status, age, gender, educational attainment, and insurance status (Table 4). The multivariable logistic regression model was found to be a statistically significant predictive model (F Value: 11.42, p < .0001). In addition to the overall model, each independent variable was statistically significant. In this adjusted model, Non-Hispanic Black, and Mexican American and other Hispanic participants have 1.63 and 1.70 times, respectively, the odds of receiving combined mental health professional and medication therapy versus other therapies than non-Hispanic White participants. Participants with comorbid CMetS and depressive symptomology have 28% lower odds (OR = 0.72, CI 0.493–1.041) of receiving combined mental health professional and medication therapy versus other therapies than participants with depressive symptomology only. Participants with CMet symptomology only have significantly higher odds (OR = 3.06, CI 2.045–4.575) of receiving combined mental health professional and medication therapy versus other therapies than participants with depressive symptomology only.

**Table 4:** Adjusted odds ratios of probability of receiving mental health treatment among adults with depressive and metabolic symptomology.

	Adjusted Odds Ratio	95% Confidence Limits	
**Race/Ethnicity** (reference category: Non-Hispanic White)			
Non-Hispanic-Black	1.638	1.238	2.167
Mexican American & Other Hispanic	1.704	1.254	2.315
Other Race and Multi-race	1.471	0.979	2.210
**Conditions** (reference category: Depressive Symptoms only)			
Cardiometabolic Only	3.059	2.045	4.575
Cardiometabolic and Depressive Symptoms	0.717	0.493	1.041
**Age** (reference category: 18–39 years)			
40–59	0.834	0.596	1.167
60+	1.531	0.984	2.382
**Gender**			
Male	1.479	1.134	1.931
**Insurance** (reference category: Private Insurance)			
No Insurance	1.780	1.105	2.866
Medicare	0.363	0.233	0.566
Medicaid	0.402	0.279	0.579
Other State Sponsored Plans	0.382	0.224	0.650
**Education** (reference category: College graduate or above)			
Less than High School Graduate	1.976	1.317	2.965
High School Graduate or General Education Development	2.372	1.606	3.504
Some College or Associate Degree	1.269	0.866	1.858

## Discussion

Overall, roughly two-thirds of all Americans with only depressive symptoms received no mental health treatment at all. Sixty-one percent of NH White persons received no treatment. More than three out of four Hispanic and NH Black persons with only depressive symptoms received no mental health treatment at all. A significant proportion of individuals with co-occurring CMetS and depressive symptomology were also untreated (did not receive either medication or mental health counseling).

Adjusted regression results show that persons with both CMetS symptoms and DD symptoms received more mental health treatment and specifically more mental health medications than people with only depressive symptoms. This may be due to having more frequent visits with healthcare professionals, an association previously documented for preventive services [20]. Also, it is possible that the mental health counseling and medications were utilized for treatment of alternate mood disorders. For example, common depression medications such as selective serotonin reuptake inhibitors (SSRI) and serotonin-norepinephrine reuptake inhibitors (SNRIs) are first-line medications for the treatment of panic disorder/agoraphobia (PDA), generalized anxiety disorder (GAD), and specific phobias [21].

Depression has been found to be an important predictor of adverse cardiac outcomes, both as a driver of behavioral risks (obesity, smoking, cholesterol, etc.) and independent of other known risk factors. Research suggests that depression and cardiometabolic risk often co-occur, exacerbating risk for adverse cardiometabolic outcomes [22, 23]. Our findings demonstrated that receiving no mental health treatment was the most common pattern across all racial/ethnic groups. Additionally, NH-White participants were more likely to receive medication only treatment, or to receive both medication and mental health professional treatment compared to other racial/ ethnic groups.

Persons experiencing comorbid depression and cardiometabolic disease are commonly prescribed psychotropic medications to treat depressive symptoms [24] without involving mental health professionals. While found to be effective in reducing depressive symptoms, some psychotropic medications have been found to be associated with adverse changes in cardiometabolic markers [22–24]. There is no clear explanation for our finding that despite the NH Black population having a higher CMetS prevalence [5], the NH Black participants in our study were more likely to receive mental health professional only therapy. This could represent greater provider concern for adverse cardiometabolic effects in Black patients, or implicit bias among providers, or perhaps patient preferences. Regardless of race / ethnicity, cardiometabolic screening and monitoring are recommended for patients with known cardiometabolic risk factors when medications are prescribed to treat depression [25, 26]. However, studies indicate wide variation in cardiometabolic screening and monitoring practices for patients prescribed antidepressant medications [27, 28].

Timely and proper treatment of mental health disorders is vital to optimal health outcomes as individuals with chronic disease are twice as likely to have mood and anxiety disorders compared to individuals free of disease [29, 30]. Among individuals with type 2 diabetes, for example, high glucose may contribute to anxiety and depression [31]. These findings are of particular importance to the Hispanic population, given the heavy diabetes prevalence burden within this population [32]. The current study demonstrated that Mexican American participants were most likely among all subjects to receive no mental health treatment. The 2023 Kaiser Family Foundation Racism, Discrimination, and Health Survey found that cost concerns and scheduling access were issues across all racial-ethnic groups, but that Hispanic, Black, and Asian adults reported additional challenges–specifically, *“finding a provider who can understand their background and experiences”* [33]. Disparate barriers to treatment may reflect both implicit and explicit biases of providers, patient preferences, system barriers, and / or mistrust, miscommunication, or other dysfunction in the provider-patient dyad [34]. System-level barriers can include a dearth of Spanish-language professionals and linguistically appropriate services, or a geographic maldistribution of services. For example, VanderWielen et al. found that primary care providers practicing in predominantly African American neighborhoods (or in rural areas) were much less likely to have behavioral health professionals nearby [35].

Adequate treatment of depression has been demonstrated to improve specific CMetS risk factors, including improved control of diabetes [36] and may also show higher efficacy in risk factor self-management. Treatment for mental health may improve cardiometabolic outcomes, as patients who use psychotropic drugs, for example, tend to have better-controlled hypoglycemia [36]. Individuals with mental illness have a greater risk of premature mortality caused by increased cardiovascular disease, which is similar to the increased cardiovascular risks associated with the metabolic dysregulations that lead to CMetS [37]. A cohort study linking National Death Index data to NHANES demonstrated a higher risk of all-cause mortality as well as cardiovascular deaths among adults with moderate to severe depressive symptoms [38].

Approximately 16 percent of our study population reported having recent depressive symptomology. As demonstrated in previously reported estimates, this relatively low level of depressive symptomology is likely an underreporting of the conditions [39]. People with mental illness may not have the necessary tools to adequately manage their blood pressure, body weight, blood sugar levels, and overall cardiovascular and metabolic health due to decreased access to medical care and adequate health education [40]. Disparate care is often documented in the literature across racial/ethnic, gender and socioeconomic groups. Ethnic minority persons may be at higher risk of developing mental disorders—which present barriers to effective chronic disease management—owing to social and environmental difficulties and the increased likelihood of underlying comorbid conditions [41]. Under-diagnosis, misdiagnosis, and under-treatment on the basis of race-ethnicity and implicit bias have all been well-documented [42]. In 2008, the World Health Organization predicted that major depression would climb from third to first in burden of disease by 2030 [43]. Although this rise in prevalence has been documented, there is still reason to believe its severity is underreported. Reasons for underreporting include difficulty detecting symptoms in a primary care setting, visit time constraints, and patient hesitation to discuss their emotions [39, 44]. According to the US Surgeon General’s **Mental Health: Culture, Race, and Ethnicity** supplemental report, “*major differences were found in some manifestations of mental disorders*, *idioms for communicating distress*, *and patterns of help-seeking”* [45].

Racial barriers also appear to undermine the capacity to establish a successful treatment process and to deliver high quality care [46]. While African American patients report more positive attitudes than whites regarding treatment seeking before they use mental health services, African Americans have less favorable attitudes after the actual experience of mental health treatment [47]. Our study demonstrated that NH Black participants were more likely to receive talk therapy only compared to other racial/ethnic groups. Understanding whether this is a patient choice, a provider bias, or an effect of mistrust or communication disconnects in the patient -physician dyad will require further research. Ultimately, the integration of mental health and primary care holds promise for helping individuals achieve both improved mental and physical health and efficacy in achieving desired health behaviors [48].

Limitations of our study include the cross-sectional nature of the dataset, which does not allow for causal inferences. This study relies on self-reported medical conditions; therefore, it is possible that some of the respondents might not meet the diagnostic threshold for a depressive disorder on other validated instruments. Conversely, patients who would likely meet the diagnostic threshold for a depressive disorder may not have sought treatment for symptoms, resulting in an underestimate of the frequency of comorbid depressive disorder and CMetS respondents. A history of CMetS also relied on self-report, potentially leading to under-reporting of CMetS-related conditions. The decision to provide mental health medical treatment may be predicated by coexisting CMetS complications. Also, NHANES does not include specific information on alternative or complementary treatments for mental health. Therefore, it is possible this study did not capture all mental health therapies utilized by study participants. Finally, the aggregation of racial/ethnic populations within the NHANES dataset, presents limitations to identifying disparate treatment within specific ethnic populations.

While these are all potentially important limitations, the strengths of this analysis include the use of 5 years of data in a nationally representative study population over-sampled for racial/ethnic minority populations, including sufficient numbers of minorities with comorbid DD/CMetS to produce nationally representative estimates of racial/ethnic differences. Additionally, these data demonstrate that a substantial proportion of persons with symptoms of depression in the United States remain untreated or undertreated.

## Conclusions

Great strides have been made in the efficacy of psychotropics, counseling therapies, and “non-traditional” therapies in the treatment of mental health conditions. Positive benefits of structured physical activity and other lifestyle interventions are known for both depressive symptoms and for CMetS [49, 50]. Additionally, mindfulness, meditation and other self-management therapies have shown positive outcomes in depression and CMetS risk factors [51, 52].

Further research is needed to address not just availability of mental health treatment, but all of the dimensions of access including affordability, integration in primary care, stigma-reduction, acceptability, and cultural relevance. The National Institute on Minority Health and Health Disparities has suggested a research framework for addressing such disparities that spans outcomes and levels of influence from the individual and family to the community and society. It suggests interventions that target biological, behavioral, environmental (both physical and sociocultural) and healthcare system factors [53].

Simultaneously treating and managing both mental illness and CMetS properly is complicated, and future research studies should investigate the untapped synergies in treating both concurrently. Ensuring financial access to care is foundational for achieving equitable care and outcomes, through some form of universal health coverage and parity for behavioral health care. Integration of behavioral health into primary care settings through collaborative care teams has been demonstrated to improve clinical outcomes [54]. Integrated approaches (including community health workers, peer counselors, or promotors) may also be relevant at the community level, especially in high-disparity segments of the population [55]. Therefore, to achieve favorable health outcomes, optimal treatment should address aspects of both mental health and metabolic disorders in an integrated approach, and may need to be culturally-tailored to be effective [56].

## Supporting information

S1 File Complete regression results. (DOCX)
