# Supplementary material for: Racial/ethnic differences in mental health treatment received among people with comorbid cardiometabolic and depressive symptomology: National Health and Nutrition Examination Survey, 2017 to March 2020 Pre-Pandemic
Source: PLoS One. 2025 Jan 2;20(1):e0316430. doi: 10.1371/journal.pone.0316430 (PMC11695010; doi:10.1371/journal.pone.0316430)
Supplement: S1 File — (DOCX) [file pone.0316430.s001.docx]

The SAS System 1

The SURVEYLOGISTIC Procedure

Model Information

Data Set One

Response Variable TreatType

Number of Response Levels 4

Weight Variable WTINTPRP Full sample interview weight

Model Cumulative Logit

Optimization Technique Fisher's Scoring

Variance Adjustment Degrees of Freedom (DF)

Variance Estimation

Method Taylor Series

Variance Adjustment Degrees of Freedom (DF)

Number of Observations Read 2981

Number of Observations Used 2821

Sum of Weights Read 71102584

Sum of Weights Used 67371893

Response Profile

Ordered Treat Total Total

Value Type Frequency Weight

1 0 2071 46667467

2 1 308 7992991

3 2 239 6559717

4 3 203 6151717

Probabilities modeled are cumulated over the lower Ordered Values.

NOTE: 160 observations were deleted due to missing values for the response or explanatory variables.

Class Level Information

Class Value Design Variables

race 1 -1 -1 -1

2 1 0 0

3 0 1 0

4 0 0 1

age 1 -1 -1

2 1 0

3 0 1

RIAGENDR 1 1

The SAS System 2

The SURVEYLOGISTIC Procedure

Class Level Information

Class Value Design Variables

2 -1

comorbid CMetS Onl 1 0

CMetS/Dep 0 1

Dep Only -1 -1

insurance 0 1 0 0 0

1 -1 -1 -1 -1

2 0 1 0 0

3 0 0 1 0

4 0 0 0 1

edcat 0 1 0 0

1 0 1 0

2 0 0 1

3 -1 -1 -1

Model Convergence Status

Convergence criterion (GCONV=1E-8) satisfied.

Model Fit Statistics

Intercept

Intercept and

Criterion Only Covariates

AIC 128354582 119591787

SC 128354630 119592075

-2 Log L 128354576 119591751

Testing Global Null Hypothesis: BETA=0

Test F Value Num DF Den DF Pr > F

Likelihood Ratio 24.46 15 2806 <.0001

Score 15.08 15 2806 <.0001

Wald 11.42 15 2806 <.0001

The SAS System 3

The SURVEYLOGISTIC Procedure

Type 3 Analysis of Effects

Effect F Value Num DF Den DF Pr > F

race 5.73 3 2818 0.0007

comorbid 21.35 2 2819 <.0001

age 4.37 2 2819 0.0128

RIAGENDR 8.32 1 2820 0.0039

insurance 13.91 4 2817 <.0001

edcat 9.13 3 2818 <.0001

Analysis of Maximum Likelihood Estimates

Standard

Parameter Estimate Error t Value Pr > |t| Exp(Est)

Intercept 0 1.1172 0.1028 10.87 <.0001 3.056

Intercept 1 1.8348 0.1110 16.53 <.0001 6.264

Intercept 2 2.7342 0.1277 21.41 <.0001 15.397

race 2 0.1405 0.1013 1.39 0.1657 1.151

race 3 0.1797 0.1114 1.61 0.1067 1.197

race 4 0.0329 0.1481 0.22 0.8242 1.033

comorbid CMetS Onl 0.8564 0.1318 6.50 <.0001 2.355

comorbid CMetS/Dep -0.5948 0.1243 -4.79 <.0001 0.552

age 2 -0.2629 0.1015 -2.59 0.0096 0.769

age 3 0.3443 0.1321 2.61 0.0092 1.411

RIAGENDR 1 0.1958 0.0679 2.88 0.0039 1.216

insurance 0 1.0383 0.1854 5.60 <.0001 2.824

insurance 2 -0.5507 0.1635 -3.37 0.0008 0.577

insurance 3 -0.4489 0.1280 -3.51 0.0005 0.638

insurance 4 -0.5005 0.2006 -2.50 0.0126 0.606

edcat 0 0.2352 0.1137 2.07 0.0387 1.265

edcat 1 0.4182 0.1098 3.81 0.0001 1.519

edcat 2 -0.2077 0.1006 -2.06 0.0391 0.812

NOTE: The degrees of freedom for the t tests is 2820.

The SAS System 4

The SURVEYLOGISTIC Procedure

Odds Ratio Estimates

Point 95% Confidence

Effect Estimate Limits

race 2 vs 1 1.638 1.238 2.167

race 3 vs 1 1.704 1.254 2.315

race 4 vs 1 1.471 0.979 2.210

comorbid CMetS Onl vs Dep Only 3.059 2.045 4.575

comorbid CMetS/Dep vs Dep Only 0.717 0.493 1.041

age 2 vs 1 0.834 0.596 1.167

age 3 vs 1 1.531 0.984 2.382

RIAGENDR 1 vs 2 1.479 1.134 1.931

insurance 0 vs 1 1.780 1.105 2.866

insurance 2 vs 1 0.363 0.233 0.566

insurance 3 vs 1 0.402 0.279 0.579

insurance 4 vs 1 0.382 0.224 0.650

edcat 0 vs 3 1.976 1.317 2.965

edcat 1 vs 3 2.372 1.606 3.504

edcat 2 vs 3 1.269 0.866 1.858

NOTE: The degrees of freedom in computing

the confidence limits is 2820.

Association of Predicted Probabilities and Observed Responses

Percent Concordant 68.0 Somers' D 0.368

Percent Discordant 31.2 Gamma 0.371

Percent Tied 0.7 Tau-a 0.161

Pairs 1737903 c 0.684
